# Unclassifiable senile plaques and extensive cerebral amyloid angiopathy involving spinal and bridging vessels in autopsied patients with Down syndrome

**DOI:** 10.17879/freeneuropathology-2026-9406

**Published:** 2026-06-01

**Authors:** Hiroaki Miyahara, Yuichi Riku, Kumiko Yano, Yousuke Hidaka, Daisuke Tahara, Nao Tahara, Hideyuki Moriyoshi, Akio Akagi, Jun Sone, Hideki Hashidate, Akiyoshi Kakita, Yasushi Iwasaki

**Affiliations:** 1 Department of Neuropathology, Institute for Medical Science of Aging, Aichi Medical University, Nagakute, Aichi, Japan; 2 Department of Behavioral Neurology and Neuropsychiatry, United Graduate School of Child Development, Osaka University, Suita, Osaka, Japan; 3 Department of Pathology, Niigata City General Hospital, Niigata, Niigata, Japan; 4 Department of Pathology, Brain Research Institute, Niigata University, Niigata, Niigata, Japan

**Keywords:** Down syndrome, Autopsy, Senile plaques, Alzheimer's disease, Cerebral amyloid angiopathy

## Abstract

**Background: **Individuals with Down syndrome (DS) face markedly increased risk of premature aging and age-related pathological changes, particularly Alzheimer's disease (AD)-like neuropathology. By the fourth decade of life, virtually all individuals with DS develop the hallmark AD features such as senile plaques (SPs) and neurofibrillary tangles (NFTs). The aim of this study was to characterize the topographical distribution of cerebral amyloid angiopathy, the morphology of senile plaques, and the spectrum of co-existing aging-related proteinopathies in autopsied DS patients, with reference to age-matched and elderly controls.

**Methods: **Nine autopsied DS patients (aged 0.5–68.0 years at death) were examined alongside age-matched controls. Immunohistochemical staining was performed for amyloid-β (Aβ), phosphorylated tau, α-synuclein, and phosphorylated TDP-43. In addition, silver impregnation using the Gallyas method and Congo red staining were performed. Aging-related pathologies were assessed using established criteria for NFTs, Aβ deposits, cerebral amyloid angiopathy (CAA), and other neurodegenerative changes.

**Results: **All four DS patients aged ≥ 28 years (D6–D9) showed moderate-to-severe AD neuropathological changes, whereas none of five age-matched controls (23.1–68.4 years old) did. In DS patients with AD, unclassifiable SPs were predominant, and NFTs with both 3-repeat and 4-repeat tau were observed. The distribution and progression of the latter were similar to those of sporadic AD patients. CAA was observed in three DS patients and, owing to systematic sampling, could be documented in the spinal arteries and subdural/subarachnoid bridging vessels—sites not routinely evaluated in autopsy series of sporadic CAA. All three DS cases with CAA reached Thal stage 3 CAA, contrasting with a maximum of stage 2 in CAA-positive sporadic AD and elderly control cases. Notably, two of three DS patients with CAA had a documented clinical history of subdural hemorrhage (SDH); both showed marked cerebral atrophy at autopsy, precluding definitive attribution of SDH to CAA. The high frequency of SDH suggests increased hemorrhagic risk in DS patients due to extensive vascular amyloid deposition.

**Conclusions: **This study demonstrates accelerated ADNC development in DS, with characteristic unclassifiable SPs and extensive CAA representing unique features that distinguish DS from common aging patterns. The clinical history of SDH in DS patients with CAA, together with the histological extension of CAA to subdural bridging vessels, may warrant attention when considering the vascular safety of emerging anti-amyloid therapies in this population. However, causality between CAA and SDH could not be established from the present autopsy data. These findings provide crucial insights into AD pathogenesis and highlight the importance of developing targeted therapeutic strategies while considering safety implications.

## Introduction

Down syndrome (DS), also known as trisomy 21, affects approximately 1 in 700 live births [2] and represents the most common genetic cause of intellectual disability. Beyond developmental and cognitive features, individuals with DS face markedly increased risk of premature aging and age-related pathological changes, particularly in the central nervous system [2,14].

The relationship between DS and accelerated aging is most prominently exemplified by neuropathological changes characteristic of Alzheimer's disease (AD). By the fourth decade of life, virtually all individuals with DS develop pathological hallmarks of AD, including senile plaques (SPs) and neurofibrillary tangles (NFTs). Individuals with DS face a lifetime risk of dementia of approximately 90 % [2], with a median age of clinical dementia onset of 53.8 years [19], although there is a wide range of age of onset [10]. While neuropathological changes are nearly universal, the age at which clinical symptoms emerge varies considerably among individuals. The molecular basis of these changes lies in the gene dosage imbalance created by chromosome 21 trisomy, particularly the triplication of the amyloid precursor protein (APP) gene, leading to increased amyloid-β (Aβ) production throughout life [9].

Key neuropathological changes in aging patients with DS include early SP deposition beginning in the second decade of life, progressive tau accumulation in neurons, distinctive neuroinflammation patterns, synaptic dysfunction, and neuronal loss [11,14]. These changes differ in timing from those of sporadic AD and are accompanied by unique cerebrovascular characteristics in terms of less severe atherosclerosis but frequent amyloid angiopathy [15,24].

The study of neuropathological changes in DS provides unique insights into aging and neurodegenerative diseases, as the consistent and predictable development of AD-like pathology offers opportunities to investigate the chronological sequence of pathological events and potential therapeutic targets. Recent advances in Aβ-targeting therapies for AD have opened new therapeutic possibilities for addressing AD-related neuropathological changes in DS, highlighting the clinical relevance of understanding the precise mechanisms and timelines of these pathological changes [22,50]. However, critical questions remain regarding the factors that determine clinical dementia development and potential protective mechanisms.

To address the remaining questions outlined above, we conducted a detailed histopathological analysis of autopsy cases from two institutions. Because premature aging in DS is already well established and our sample size was limited, we focused on descriptive neuropathological characterization, with particular emphasis on cerebrovascular Aβ pathology. Specifically, we aimed to characterize: (1) the topographical distribution of cerebral amyloid angiopathy (CAA), including its involvement of the anterior spinal arteries and subdural/subarachnoid bridging vessels, and its association with subdural hemorrhage; (2) the morphological features of senile plaques in DS, with comparison to previously described plaque subtypes (neuritic, diffuse, cotton-wool, coarse-grained, and bird-nest plaques); and (3) the spectrum of co-existing aging-related proteinopathies, including Aβ, hyperphosphorylated tau, α-synuclein, and phosphorylated TAR DNA-binding protein of 43 kDa (TDP-43), in DS compared with age-matched and elderly controls. We examined nine DS autopsy cases aged 0.5–68.0 years and 14 control cases using comprehensive immunohistochemistry (IHC), silver impregnation with the Gallyas method, and Congo red staining.

## Materials and methods

### Enrolled autopsied patients and their clinical information

**Figure 1: Visualization of the neuropathological aging-related pathologies in DS, AD, and control brains:**
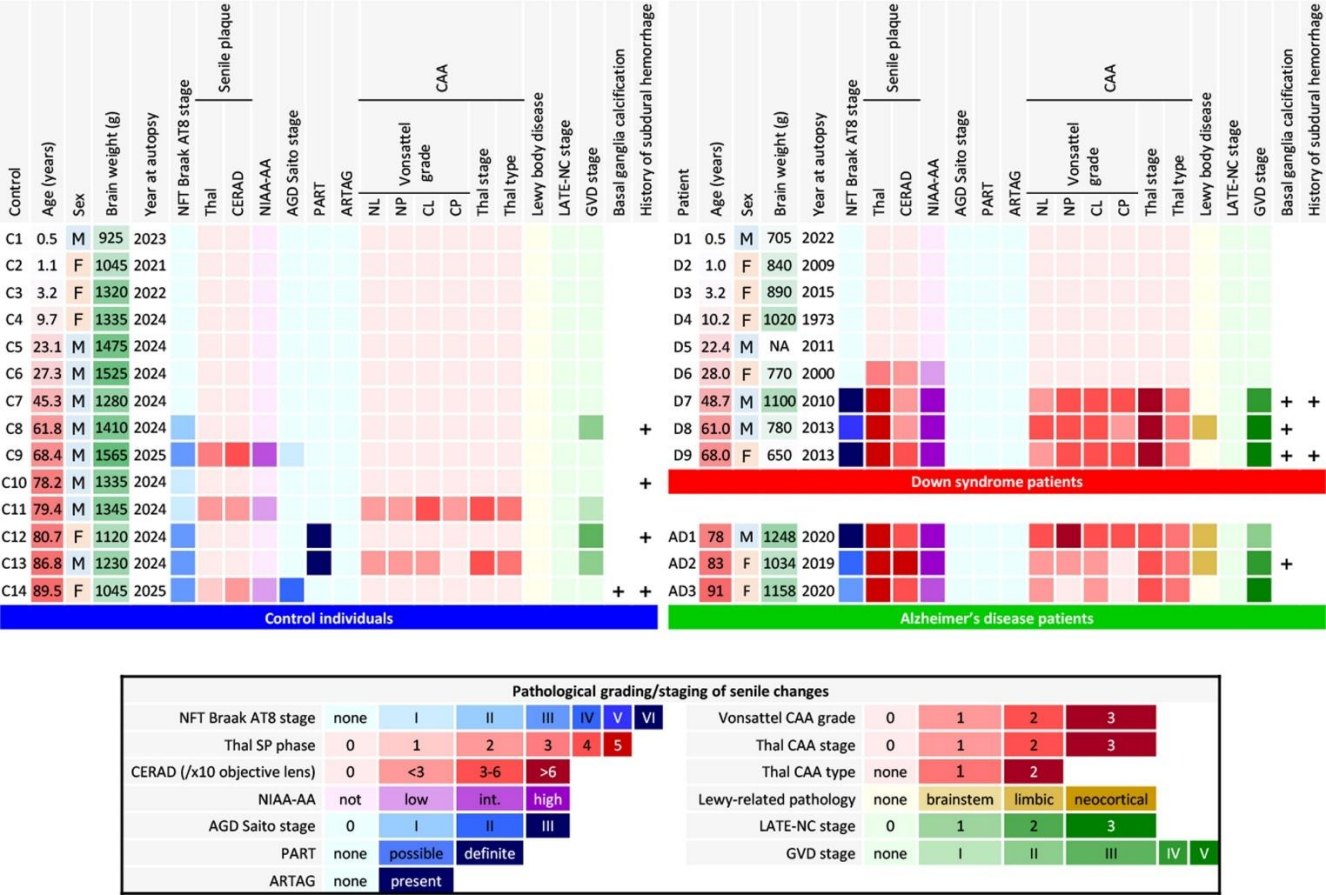
Moderate to severe AD neuropathological change was observed in DS patients aged 28.0 years and older. In age-matched control individuals (C5–C9), no comparable AD pathology was observed. Patient D6 had abundant SPs but no NFTs. Vonsattel CAA grade: neocortical parenchymal vessels (see Results for compartment-specific grades). CAA was extensive and included involvement of the anterior spinal arteries in patients D7–D9. Limbic-type LBD was observed in patient D8, AD1, and AD2. No AGD, PART, ARTAG, or LATE-NC was observed in DS, AD, or age-matched control individuals.

Nine autopsied patients with DS (D1–D9), archived between 1973 and 2022 at the Aichi Medical University Karei Ikagaku Brain Resource Center (AKBRC) and the Brain Research Institute of Niigata University, were included in this study. All the patients were Japanese and had been diagnosed with DS by karyotyping. Subject ages ranged from 0.5 to 68.0 years. Nine age-matched forensic individuals (C1–C9) and 5 forensic individuals aged over 70 years without cognitive decline (C10–C14) served as normal controls. To evaluate the characteristics of the SPs, three sporadic AD patients (AD1–AD3) were also included in this study. Detailed demographic and clinical information for all enrolled cases is summarized in **Figure 1**. Autopsies were performed between 1973 and 2022, and the interval from autopsy to the present histopathological re-evaluation ranged from 3 to 52 years. Immunohistochemical analyses for the present study were performed between 2025 and 2026 using sections newly cut from archived formalin-fixed paraffin-embedded blocks, which had been preserved under standardized archival conditions throughout this period.

### Histological and immunohistochemical analyses

Autopsied specimens were fixed with 20 % buffered formalin and embedded in paraffin. In this study, the left hemisphere was consistently used as the primary site for histological and immunohistochemical analysis in all cases. Histological examinations were performed using sections processed by hematoxylin‒eosin (HE), Klüver‒Barrera (KB), silver-impregnation with the Gallyas method, and Congo red staining. Immunohistochemical examinations were performed as previously described [16]. Briefly, sections were incubated overnight with the following primary antibodies: anti-α-synuclein antibody (polyclonal; S3062; Sigma-Aldrich, MO, USA; 1:20,000, after heat-induced antigen retrieval and formic acid pretreatment), anti-CD68 (monoclonal; clone PG-M1; M0876; DAKO, Glostrup, Denmark; 1:200, after heat-induced antigen retrieval and trypsin pretreatment), anti-GFAP (monoclonal; clone 6F2; M761; DAKO, Glostrup, Denmark; 1:500, pretreated by heat antigen retrieval), anti-human amyloid-β (11–28) (Aβ₁₁₋₂₈) antibody (monoclonal; clone 12B2; #10027; IBL, Gunma, Japan; 1:1,000, pretreated with formic acid), anti-human amyloid-β (1–40) (Aβ_1–40_) antibody (polyclonal; #18580; IBL, Gunma, Japan; 1:800; pretreated by formic acid), anti-human amyloid-β (1–42) (Aβ_1–42_) antibody (polyclonal; #18582; IBL, Gunma, Japan; 1:400; pretreated with formic acid), anti-Iba1 antibody, phospho-tau (Ser202, Thr205) antibody (monoclonal, clone AT8; MN1020; Thermo Scientific, IL, USA; 1:5,000), anti-phospho TDP-43 (pS409/410) antibody (polyclonal; TIP-PTD-P07; CosmoBio, Tokyo, Japan; 1:4,000; after heat-induced antigen retrieval and formic acid pretreatment ), anti-tau (3-repeat isoform RD3) antibody (monoclonal; clone 8E6/C11; #05-803; Upstate, Syracuse, NY, USA; 1:2,500, after heat-induced antigen retrieval and formic acid pretreatment ), and anti-tau (4-repeat isoform RD4) antibody (monoclonal; clone 1E1/A6; #05-804; Millipore, Temecula, CA, USA; 1:500, after heat-induced antigen retrieval and formic acid pretreatment ). The sections were then washed with phosphate-buffered saline 5 times for 5 minutes each and incubated with secondary antibody (Histofine Simple Stain MAX PO [MULTI]; Nichirei Bioscience Inc., Tokyo, Japan) for 1 hour. The sections were visualized using 3,3'-diaminobenzidine (DAB; DAB Tablet; FUJIFILM, Osaka, Japan), and Mayer's hematoxylin solution was used as a counterstain.

The number, percentage of neuritic plaques, average size, area occupied, and mean gray value (as a measure of Aβ_11–28_ immunoreactivity) of the SPs in DS patients with AD (DS+AD) and in sporadic AD patients were measured using ImageJ Ver. 1.53t software (https://imagej.net/ij/) on Aβ_11–28_-immunostained sections of the middle temporal gyrus taken at x10 objective magnification (Figure 4), as previously described [29].

### Assessment of aging-related pathologies

Aging-related or comorbid pathological changes were assessed using published pathological criteria for NFTs [5], Aβ deposits [28,44], argyrophilic grain disease (AGD) [34] and Lewy body disease (LBD) [27]. Comprehensive assessment of the Alzheimer’s pathology was dependent on the international criteria suggested by the Montine TJ et al [30]. Primary age-related tauopathy (PART) [8] and aging-related tau astrogliopathy (ARTAG) [23] were assessed using their respective diagnostic criteria. The severity of vessel-wall involvement in cerebral amyloid angiopathy (CAA) was graded using the Vonsattel scale [47], assessed separately for parenchymal and leptomeningeal vessels in both the neocortex (middle frontal, middle temporal, and occipital cortex) and the cerebellum. The topographical expansion and biochemical subtype of CAA were classified using the Thal CAA stage [42] and Thal CAA type [43], respectively. Aβ_11–28_ immunostaining was performed on sections from the neocortex, allocortex, hippocampus, basal ganglia, thalamus, white matter, brainstem, and cerebellum to evaluate the topographical distribution of CAA according to the Thal CAA staging system [42]. The Vonsattel CAA grade displayed for each case in Figure 1 represents the grade of leptomeningeal vessels in the neocortex, which were selected as the representative compartment because leptomeningeal involvement is generally the most diagnostically informative for CAA severity. Region- and compartment-specific grades for all four assessed compartments are reported in the Results section. Phosphorylated TDP-43-positive neuronal cytoplasmic inclusions in the limbic system were scored using neuropathologic criteria for limbic-predominant age-related TDP-43 encephalopathy neuropathological change (LATE-NC) [31]. The expansion of granulovacuolar degeneration (GVD) was assessed using GVD staging [41]. The presence and severity of mineralization were assessed on HE-stained sections of the lentiform nucleus, the cerebellar white matter, and the dentate nucleus, with additional Perls staining to evaluate the contribution of iron. The term *mineralization* is used throughout in preference to *calcification*, reflecting the mixed calcium- and iron-containing nature of these vascular deposits in DS [44]. Regions used for each pathological staging/criteria system are shown in **Table 1**. Statistical comparisons used Fisher's exact test for binary categorical data, the Fisher-Freeman-Halton exact test for ordinal categorical data, and the Mann-Whitney U exact test for ordinal Vonsattel grades. All p-values are exact two-sided and are presented as exploratory given the small group sizes.

**Table 1:** Regions used for each pathological staging/criteria system

Staging system	Staining/Antibody	Regions assessed
Braak NFT stage	AT8	Transentorhinal cortex, entorhinal cortex, hippocampus (CA1/subiculum), temporal/occipital/frontal neocortex
Thal Aβ phase	Aβ_11–28_	Neocortex (1) → allocortex/hippocampus (2) → diencephalon/striatum (3) → brainstem (4) → cerebellum
CERAD	Gallyas/Aβ_11–28_	Frontal, temporal, parietal neocortex
Thal CAA stage	Aβ_11–28_	Stage 1: Neocortex Stage 2: Hippocampus, allocortex, cerebellum Stage 3: Basal ganglia, thalamus, white matter, brainstem
Thal CAA type	Aβ_11–28_	Cortical capillaries on Aβ_11–28_ in frontal/temporal/occipital cortex (Type 1: capillary-positive; Type 2: capillary-negative)
Vonsattel grade	Aβ_11–28_	Leptomeningeal and cortical vessels of neocortex and cerebellum
GVD stage	p-TDP-43	Hippocampus (CA1–CA4, subiculum), entorhinal cortex, neocortex
LATE-NC stage	p-TDP-43	Amygdala, hippocampus, middle frontal gyrus
ARTAG	AT8	Subpial, perivascular, white matter, gray matter regions of frontal/temporal lobes and amygdala
AGD	4R-tau/Gallyas	Amygdala, ambient gyrus, hippocampus
PART	AT8	Hippocampus, entorhinal cortex without significant Aβ
LBD	α-synuclein	Brainstem (DMV, LC, SN), amygdala, hippocampus, cingulate, neocortex

Aβ, amyloid-β; AGD, argyrophilic grain disease; ARTAG, aging-related tau astrogliopathy; AT8, monoclonal antibody against phosphorylated tau (Ser202/Thr205); CA1–CA4, cornu Ammonis subfields 1–4 of the hippocampus; CAA, cerebral amyloid angiopathy; CERAD, Consortium to Establish a Registry for Alzheimer's Disease; DMV, dorsal motor nucleus of the vagus; GVD, granulovacuolar degeneration; IHC, immunohistochemistry; LATE-NC, limbic-predominant age-related TDP-43 encephalopathy neuropathological change; LBD, Lewy body disease; LC, locus coeruleus; NFT, neurofibrillary tangle; PART, primary age-related tauopathy; p-TDP-43, phosphorylated TDP-43; SN, substantia nigra; 4R-tau, 4-repeat tau.

## Results

### Clinical and autopsy information in DS patient and control individuals

As shown in Figure 1, DS patients died at ages ranging from 0.5 to 68.0 years. The DS brain weights ranged from 650 to 1,100 grams and were lower than those of the control individuals at all ages. A clinical history of subdural hemorrhage (SDH) was documented for patients D7 and D9. Causes of death were congenital heart disease and infection in the infantile cases and aspiration pneumonia in the adult cases. The two patients with SDH (D7, D9) had no documented head trauma, and SDH did not contribute to death. Calcification of the globus pallidus was observed in patients D7–D9, as well as in one elderly control (C14) and one sporadic AD patient (AD2). Moderate to severe AD neuropathological change (ADNC) was observed in all DS patients aged 28.0 years and older (4/4 cases: D6–D9). In contrast, none of the age-matched control individuals (C5–C9, aged 23.1–68.4 years) showed AD pathology of comparable severity. Given the small sample size (n = 9 in each group), these observations are presented descriptively rather than as the result of formal statistical comparison. Patient D6 exhibited abundant and extensive SPs but no NFT. CAA, including in the anterior spinal arteries and subdural bridging blood vessels, were extensive in DS patients aged 48.7 years and older. Limbic-type LBD was observed in patients D8, AD1, and AD2. AGD, PART, ARTAG, and LATE-NC were observed in control individuals but not in DS patients. Formal cognitive assessment was not available for any of the DS patients, owing to early developmental stage in the infantile and pediatric cases (D1–D5) and to limited clinical documentation combined with the baseline intellectual disability of DS in the adult cases (D6–D9).

### ADNC in DS brains

**Figure 2: Neuropathological characteristics of SPs in DS brains:**
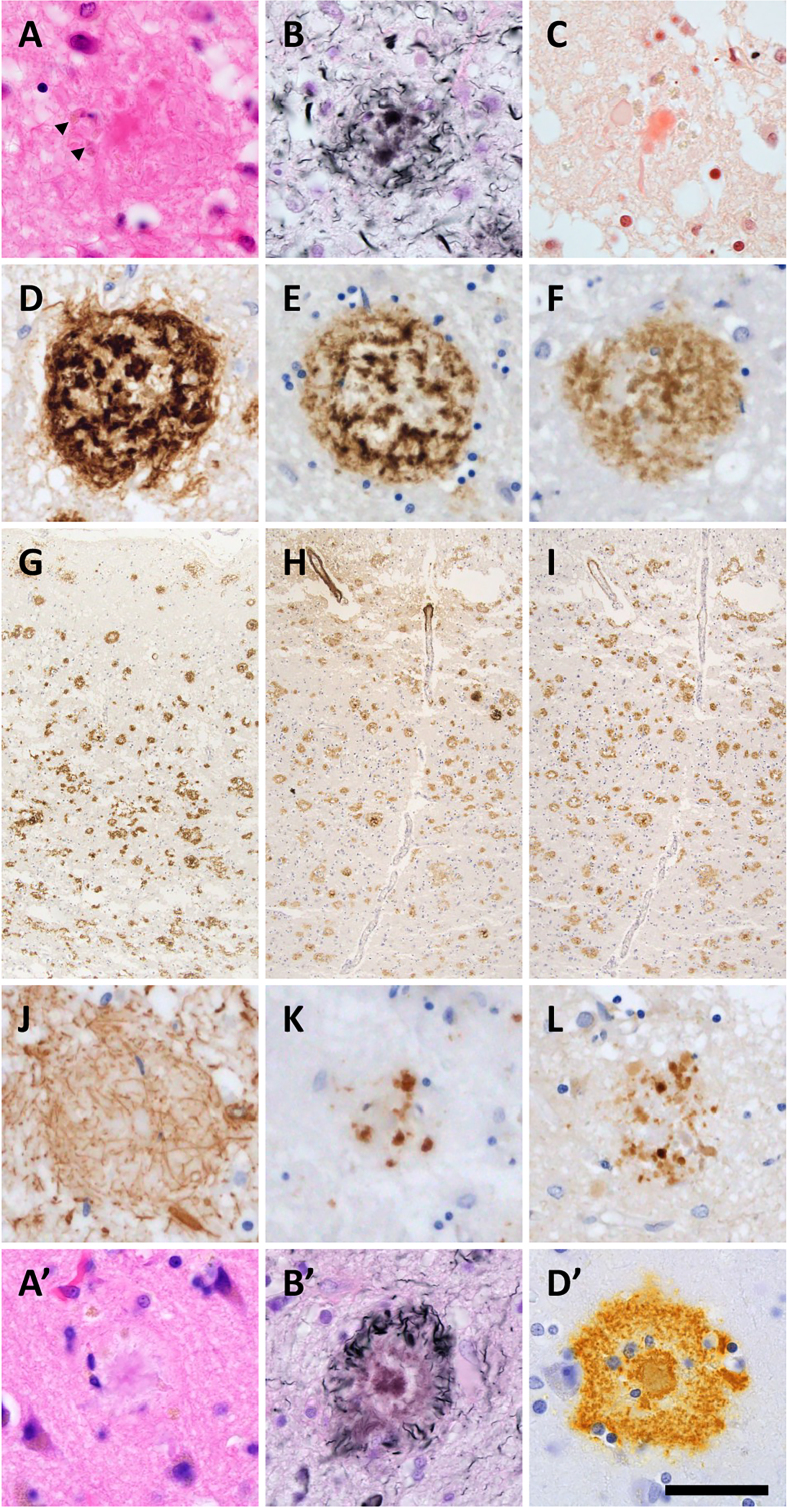
(**A**) Tissue distortion and yellow-brown deposits (arrowheads) were observed in SP centers in DS by HE staining. (**B, C**) Silver impregnation and Congo red staining demonstrated fibrillary and spheroidal amyloids. (**D, G**) Anti-Aβ_11–28_ staining revealed relatively large, Aβ-depleted pores and ill-defined borders compared with those of classic neuritic plaques. (**E, F, H, I**) The SPs in DS patients were immunoreactive for both Aβ_1–40_ and Aβ_1–42_ antibodies. (**J**) GFAP-positive disrupted processes were shown. (**K**) CD68-positive cells, representing macrophages and/or activated microglia, were observed in SP centers. (**L**) Fibrillary and spheroidal structures immunoreactive for APP were found. For comparison, representative neuritic plaques from a sporadic AD case are shown in panels A', B', and D' (HE, Gallyas silver impregnation, and Aβ_11–28_ immunostaining, respectively), demonstrating the well-defined dense Aβ cores and dystrophic neurites typical of classic neuritic plaques. (A, A') HE staining, (B, B') silver-impregnation with the Gallyas method, (C) Congo red staining, (D, D', G) anti- Aβ_11–28_ staining, (E, H) anti-Aβ1-40 staining, (F, I) anti- Aβ_1–42_ staining, (J) anti-GFAP staining, (K) anti-CD68 staining, and (L) anti-APP staining. Bar: 50 μm for (A–F, J–L, A', B', D') and 400 μm for (G–I).

**Figure 3: Distribution of SPs in DS patients and comparison with Thal phases:**
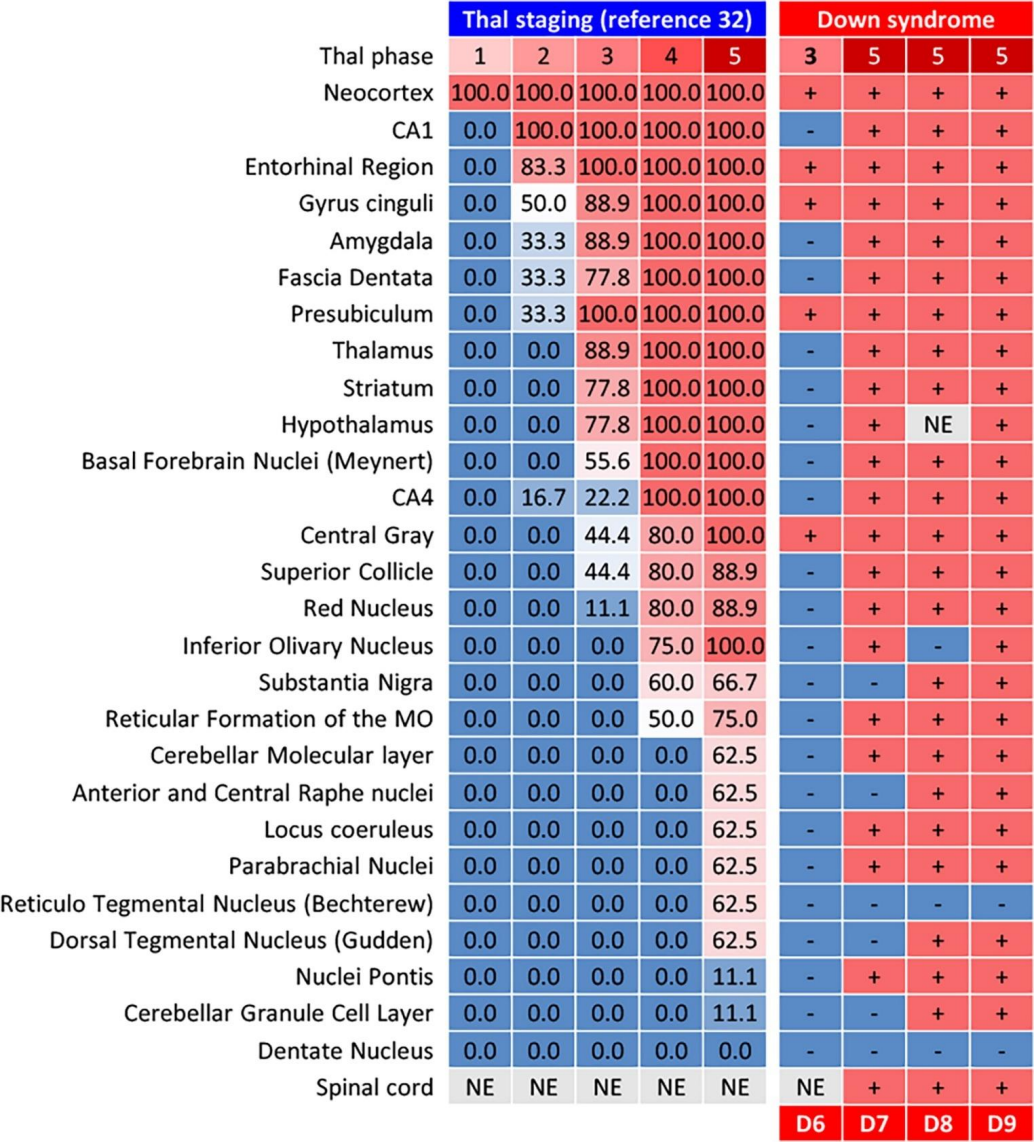
SPs in DS patients were distributed in accordance with Thal phases.

**Figure 4: Image analysis and comparison of the SPs between DS and AD patients:**
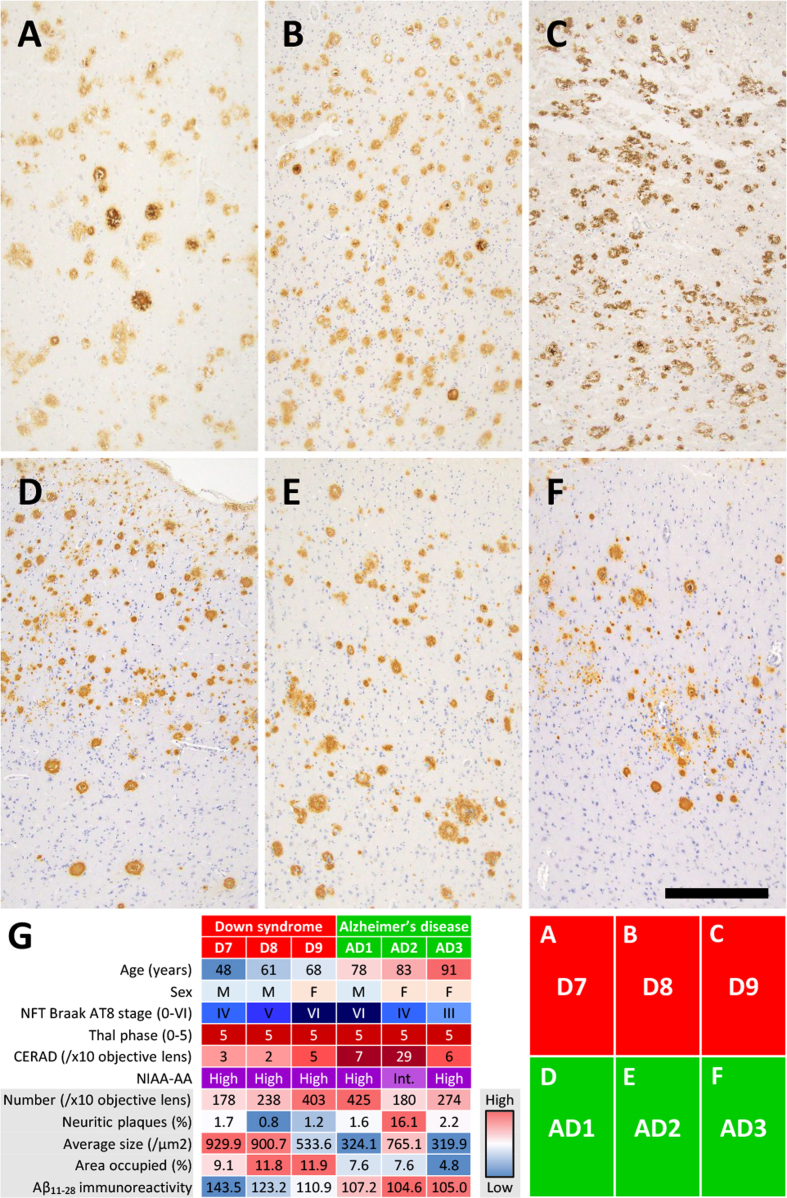
(A–F) Aβ_11–28_ IHC images of the middle temporal gyrus at x10 objective magnification. Images A–C were taken from DS patients and images D–F were taken from AD patients. (G) Heatmap of the image analysis comparing DS+AD patients (D7–D9, n = 3) and sporadic AD patients (AD1–AD3, n = 3). Each cell shows the value for an individual case, color-coded relative to the range across all six cases (red = high, blue = low). Demographic and pathological features (age, sex, NFT Braak AT8 stage, Thal Aβ phase, CERAD score, NIA-AA ADNC level) are shown for reference in the upper rows. The lower rows show the five image analysis parameters: number of SPs per ×10 objective field, percentage of neuritic plaques, average plaque size (μm²), area occupied by plaques (%), and mean gray value for Aβ_11–28_ immunoreactivity (higher values indicate weaker staining). The DS+AD cases (n = 3) showed lower proportions of neuritic plaques, larger average plaque sizes, greater areas occupied by plaques, and weaker Aβ_11–28_ immunoreactivity than the sporadic AD cases (n = 3); these are presented as exploratory observations, given the small group sizes. (A–F) Anti- Aβ_11–28_ staining. Bar: 400 μm for (A–F).

The majority of SPs in DS+AD patients presented central tissue distortion and yellow-brown deposits on HE staining, with fewer classic neuritic plaques (**Figure 2A**). Silver-impregnation with the Gallyas method and Congo red staining revealed fibrillary and spheroidal amyloids (**Figure 2B, C**). Anti-Aβ_11–28_ staining revealed relatively large, Aβ-devoid pores and ill-defined borders compared with those in classic neuritic plaques (**Figure 2D**), and the SPs in DS were visualized by both Aβ_1–40_ and Aβ_1–42_ IHC (**Figure 2D–I**). GFAP-positive disrupted processes were observed in the SPs (**Figure 2J**), and CD68-positive cells, consistent with macrophages and/or activated microglia, were observed in the center of the SPs (**Figure 2K**). Fibrillary and spheroidal structures immunoreactive for APP were found in the center of the SPs (**Figure 2L**). SPs in DS+AD had less in common with the typical neuritic, diffuse, cotton-wool, and coarse-grained plaques. Interestingly, profiles of SPs in our DS+AD individuals also differed from those of bird-nest plaques [18], which were reported to be seen in the DS+AD individuals. Due to above reasons, our DS+AD plaques were estimated as unclassifiable plaques (**Table 2**). Furthermore, SPs in the gray matter of the spinal cord were observed in all DS patients aged 48.7 years or older but not in control individuals. SPs in DS+AD patients extended in the same pattern as those in common AD patients (**Figure 3**) but appeared and spread rapidly starting at approximately 30 years of age (**Figure 1**). Image analysis of Aβ_11–28_-immunostained sections from the middle temporal gyrus (**Figure 4A–F**) provided quantitative support for these morphological observations (**Figure 4G**). Compared with sporadic AD patients (n = 3: AD1–AD3), DS+AD patients (n = 3: D7–D9) showed a comparable number of SPs per × 10 objective field (mean 273 vs. 293), but a markedly lower proportion of neuritic plaques (mean 1.2 % vs. 6.6 %), a larger average plaque size (788 vs. 470 μm²), a greater area occupied by plaques (10.9 % vs. 6.7 %), and a higher mean gray value for Aβ_11–28_ staining (126 vs. 106), the latter indicating weaker immunoreactivity. Given the limited number of cases (n = 3 in each group), these differences are presented descriptively rather than as the result of formal statistical comparison.

**Figure 5: Aging-related pathologies other than senile plaques in DS brains:**
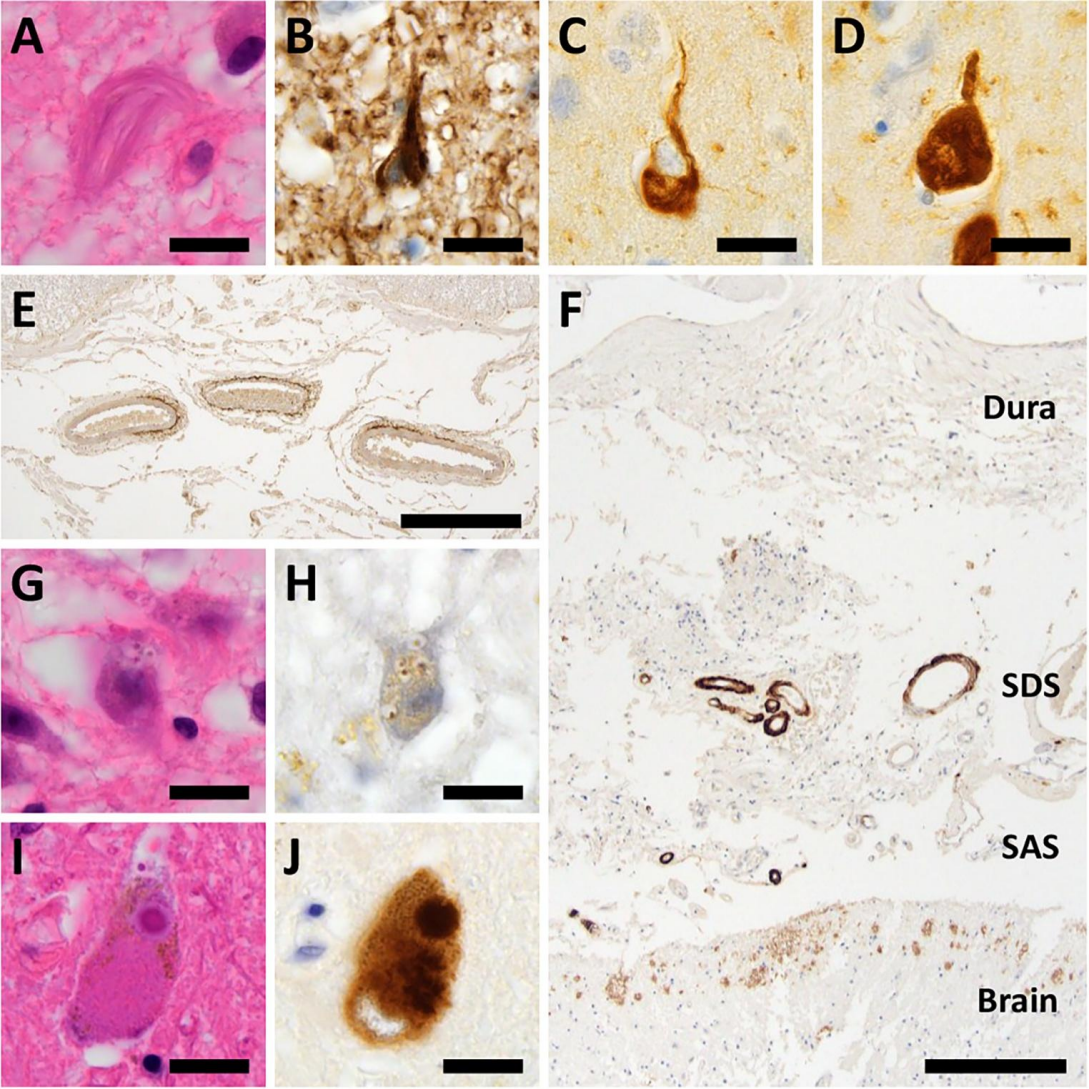
(**A, B**) NFTs in the temporal lobe in patients D7–D9 were visualized by phosphorylated tau (B). (**C, D**) These NFTs also showed immunoreactivity against both 3-repeat (C) and 4-repeat (D) tau. (**E**) Anterior spinal cord arteries in DS patients were affected by amyloid deposits. (**F**) Aβ deposits were observed in bridging vessels of the subdural and subarachnoid spaces in DS patients. (**G, H**) Anti-phosphorylated TDP-43 IHC labeled granulovacuolar degeneration in the hippocampus of DS patients (H). (**I, J**) Lewy bodies in the substantia nigra in patient D8 were visualized by α-synuclein IHC (J). (A, G, I) HE staining, (B) anti-AT8 staining, (C) anti-RD3 staining, (D) anti-RD4 staining, (E, F) anti-Aβ staining, (H) anti-phospho-TDP-43 staining, and (J) anti-α-Synuclein staining. Bar: 20 μm for (A–D, G–J), 400 μm for (E), and 250 μm for (F). SAS: subarachnoid space, SDS: subdural space.

In DS+AD patients, NFTs, visualized by antibodies against hyperphosphorylated tau, also appeared and expanded in the same pattern as in those in sporadic AD patients (**Figure 5A, B**) and showed immunoreactivity against both 3-repeat and 4-repeat tau (**Figure 5C, D**), which is consistent with AD. PART, in which the appearance of NFTs preceded that of SPs, was not observed among DS and AD patients.

ADNC, assessed by the combination of NFTs and SPs, was moderate to severe in all DS patients aged 28.0 years and older (4/4 cases: D6–D9). In contrast, no comparable AD pathology was observed in age-matched control individuals within the same age range (0/5 cases: C5–C9) (**Figure 1**). Given the small sample size, these observations are presented descriptively rather than as the result of formal statistical comparison.

**Table 2:** Comparison of SPs in DS with various SPs

Neuritic	Diffuse	Cotton-wool	Coarse-grained	Bird-nest	Regions assessed
**References**	[45]	[49]	[25]	[4]	[18]	
**Associate with**	Sporadic AD	PSEN1 mutation	APOE ε4 allele	DS	DS	
**Classical morphology**	core and crown	boundary unclear	cotton–wool like	coarse granules	bird-nest like	coarse granules
**Identifiable by HE staining?**	yes	no	yes	difficult	yes	difficult
**Visualized with S** **ilver impregnation?**	yes	no/weak	no/weak	yes	yes	yes
**Visualized with ** **Congo red staining?**	yes	no	no	yes	yes	yes
**Predominance of Aβ_1–40_ or Aβ_1–42_**	both	Aβ_1–40_	Aβ_1–40_	Aβ_1–40_	Aβ_1–40_	both
**Macrophage** **/microglia** ** infiltration**	+	–/+	–/+	+	+	+
**Astrocyte infiltration**	+	–	–	–/+	+	–
**Immunoreactivity against APP**	+	–/+	–/+	+	+	+

Aβ: amyloid–β, AD: Alzheimer’s disease, APP: amyloid precursor protein, DS: Down syndrome, HE: hematoxylin and eosin, SPs: senile plaques

### CAA in DS brains

Consistent with previous neuropathological reports [15], CAA was observed in DS patients aged 48 years and older in our cohort, supporting the well-established age-related emergence of amyloid angiopathy in DS. Aβ deposition was observed in numerous vessels on the brain surface and parenchyma from large to capillary vessels, and was labeled by both Aβ_1–40_ and Aβ_1–42_; hence, DS patients were categorized as CAA type I. Strikingly, CAA in DS patients was observed not only in the cerebrum and cerebellum but also in the anterior spinal arteries and bridging vessels of the subdural and subarachnoid spaces (**Figure 5E, F**). All three DS+CAA patients (D7–D9) reached Thal CAA stage 3, based on the presence of Aβ-immunoreactive vascular deposits in deep brain regions (basal ganglia, thalamus, and brainstem) in addition to neocortical, hippocampal, and cerebellar involvement. Group-wise summary data of all CAA scores in CAA-positive cases of each group, with statistical comparisons, are presented in **Table 3**. All 3 DS+CAA patients reached Thal CAA stage 3, whereas none of the CAA-positive sporadic AD or elderly control cases exceeded stage 2 (Fisher-Freeman-Halton exact test, p = 0.100 for both comparisons; the minimum two-sided p-value achievable for these marginal configurations). Thal CAA type 1 predominated in all three groups (DS+CAA 3/3, sporadic AD 2/3, elderly controls 2/2; p = 1.000). Vonsattel grades by compartment showed no significant between-group differences (Mann-Whitney U exact, all p ≥ 0.200; **Table 3**), with cerebellar leptomeningeal vessels uniformly grade 2 in DS+CAA cases. Across all three DS+CAA cases, capillary CAA consistently co-occurred with arteriolar and/or venular involvement, and no region with capillary-only CAA was identified.

The frequency of CAA differed substantially among the four groups examined. CAA was observed in three of nine DS patients (33.3 %; cases D7–D9, all aged 48.7 years and older), in none of nine age-matched control individuals (0 %; C1–C9, aged 0.5–68.4 years), in two of five elderly control individuals over 70 years of age (40 %; C11 and C13), and in 3 of 3 sporadic AD patients (100 %; AD1–AD3, aged 78–91 years). When restricted to DS patients within the age range at which CAA was observed in our cohort (≥ 48.7 years), all three DS patients were CAA-positive, whereas none of the three age-range-matched control individuals (C7–C9, aged 45.3–68.4 years) showed CAA. Although this categorical contrast is striking, the small sample sizes of both groups (n = 3 each) limit the statistical detection power: Fisher's exact test yielded p = 0.100, which represents the minimum achievable two-sided p-value for this 2×2 configuration with these marginal totals. The frequency in DS at this age range was comparable to that in elderly control individuals over 70 years (3/3 vs. 2/5; p = 0.196) and in sporadic AD patients (3/3 vs. 3/3; p = 1.000). These comparisons are presented as exploratory observations. Together, these findings suggest that CAA in DS appears at substantially younger ages than in cognitively intact individuals while reaching a frequency comparable to that observed in elderly controls and sporadic AD patients. Notably, Aβ deposition was histologically observed in subdural bridging vessels in DS+CAA patients (**Figure 5F**), an unusual extracerebral location for CAA. The relationship between this finding and the clinical history of SDH is detailed below.

**Table 3:** Group-wise summary of CAA scores in CAA-positive cases

Score	① DS+CAA (n = 3)	② Sporadic AD (n = 3)	③ Controls with CAA (n = 2)	p-value (① vs ②)	p-value (① vs ③)
Thal CAA stage*					
Stage 1	0 (0%)	0 (0%)	0 (0%)	0.100	0.100
Stage 2	0 (0%)	3 (100%)	2 (100%)		
Stage 3	3 (100%)	0 (0%)	0 (0%)		
Thal CAA type**					
Type 1 (capillary +)	3 (100%)	2 (67%)	2 (100%)	1.000	1.000
Type 2 (capillary −)	0 (0%)	1 (33%)	0 (0%)		
Vonsattel grade***	D7, 8, 9	AD1, 2, 3	C11, C13		
Neocortex, leptomeningeal	1, 2, 1	2, 1, 1	1, 1	1.000	1.000
Neocortex, parenchymal	2, 2, 2	3, 1, 0	1, 1	0.700	0.200
Cerebellum, leptomeningeal	2, 2, 2	2, 1, 1	2, 1	0.400	0.800
Cerebellum, parenchymal	2, 1, 2	2, 0, 0	1, 0	0.400	0.400

*: Fisher–Freeman–Halton exact test, **: Fisher exact, ***: Mann-Whitney U (exact, two-sided)

### Hemorrhagic alterations in DS brains

A clinical history of SDH prior to death was documented in 2 of the 9 DS patients (D7 and D9), both of whom had advanced CAA. By contrast, in the control cohort, a history of SDH was documented in four of the 14 individuals (C8, C10, C12, and C14), none of whom had CAA, whereas the two CAA-positive controls (C11 and C13) had no history of SDH. In the sporadic AD group, one of the three patients (AD2) had a history of SDH and was also CAA-positive. Thus, in our cohort, all SDH events in DS patients occurred in CAA-positive cases, whereas in control individuals SDH and CAA were observed in distinct individuals, suggesting that the mechanisms underlying SDH may differ between these populations. At autopsy, however, no acute or residual hematoma was identified in either case, consistent with prior resolution of the SDH. Although no documented history of head trauma was identified for either patient, the retrospective nature of this study and the limited availability of detailed clinical records preclude rigorous exclusion of antecedent traumatic events. Moreover, both patients showed marked cerebral atrophy (D7: 1100 g; D9: 650 g), which itself constitutes an established risk factor for traumatic SDH from minor head injury through bridging vein stretch. The macroscopic autopsy records of all DS patients were also reviewed for evidence of other hemorrhagic alterations; no documented evidence of intracerebral hemorrhage (ICH) or convexity subarachnoid hemorrhage (cSAH) was identified in any DS case. Furthermore, cortical superficial siderosis (cSS) or lobar cerebral microbleeds was not observed microscopically in the present study.

### GVD in DS brains

Neuronal intracytoplasmic vacuoles, which contained small granules and were visualized by antibodies against phosphorylated TDP-43, were observed in three of the nine DS patients, all three AD patients, and four of the 14 control individuals, with essentially the same or more confined distributions as those of the NFTs in the DS patients, AD patients, and control individuals (**Figure 5G, H**).

### LBD in DS brainse

Lewy bodies, visualized by antibodies agains α-synuclein, were observed in the substantia nigra and amygdala of patients D8, AD1, and AD2 (**Figure 5I, J**). These patients were neuropathologically diagnosed with limbic-type LBD (**Figure 1**).

### Associations among neuropathological alterations in DS patients

To address whether CAA was preferentially associated with ADNC rather than with other aging-related pathologies, we examined the per-case co-occurrence patterns shown in **Figure 1**.

CAA was confined to DS patients with the highest level of ADNC. All three DS+CAA patients (D7–D9) simultaneously exhibited Thal Aβ phase 5, Braak NFT stage V–VI, frequent CERAD (Consortium to Establish a Registry for Alzheimer's Disease) neuritic plaques, and "high" ADNC, whereas no DS patient below this threshold had CAA. Notably, patient D6 (28.0 years), who showed abundant senile plaques but no NFTs, lacked CAA, indicating that CAA in DS did not arise from parenchymal Aβ deposition alone (Fisher’s exact test, 3/3 vs. 0/6; p = 0.005). The CAA phenotype was uniform across the three patients (Vonsattel grade 2, Thal CAA stage 3, type 1), and A clinical history of SDH was documented only in DS+CAA patients (2/3 vs. 0/6).

GVD progressed in parallel with NFT pathology: stages III-V were restricted to the three DS patients with Braak NFT V–VI. In contrast, limbic-type LBD was observed in a single patient (D8) without apparent association with CAA stage, and AGD, PART, ARTAG, and LATE-NC were absent in all DS patients despite being present in elderly controls.

Thus, in our DS cohort, CAA and GVD were specifically linked to high-level ADNC, whereas LBD and the other aging-related tauopathies/TDP-43 proteinopathies behaved independently of ADNC severity.

### Other aging-related pathologies in DS brains

Pallidal mineralization, characterized by basophilic concentric deposits within and around small vessels on HE staining with iron co-deposition on Perls staining, was identified in three of nine DS patients (D7–D9, aged 48.7 years and older), consistent with previous reports of premature basal ganglia mineralization as a recognized feature of DS [40,48]. Comparable pallidal mineralization was also observed in one of 14 control individuals (C14, 89.5 years) and in one of three sporadic AD patients (AD2, 83 years). Both individuals were considerably older than the DS+CAA cases. These findings suggest that pallidal mineralization is not exclusive to DS but appears at a substantially younger age in DS than in non-DS individuals. No comparable mineralization was detected in the cerebellar white matter or the dentate nucleus in any DS case, nor in any age-matched control individual. AGD, PART, ARTAG, or LATE-NC were not observed in DS patients, AD patients, or age-matched control individuals; in controls over 70 years old, two cases of AGD, two cases of PART, and one case of ARTAG were neuropathologically confirmed.

## Discussion

This study examined nine autopsied DS patients aged 0.5 to 68.0 years. Moderate to severe ADNC was observed in all DS patients aged 28 years or older, whereas no age-matched control individual showed AD pathology of comparable severity within the same age range. In DS+AD patients, unclassifiable SPs were predominant and appeared rapidly starting at approximately 30 years of age. NFTs with both 3-repeat and 4-repeat tau were observed. Their distribution and progression were similar to those of sporadic AD. Owing to systematic sampling of the spinal cord and dural vasculature in our autopsy protocol — sites not routinely evaluated in standard AD/CAA series — CAA in our DS patients could be documented in the spinal arteries and subdural/subarachnoid bridging vessels. Of note, two of three DS+CAA patients had a documented clinical history of SDH prior to death. Other aging-related pathologies including AGD, PART, and LATE-NC were absent in DS patients, distinguishing them from common aging patterns.

Adult DS represents a unique model for understanding AD pathogenesis due to early-onset amyloid pathology. Autopsy studies have demonstrated that by the age of 40 years, virtually all individuals with DS exhibit SPs in their brains, markedly earlier than the onset of sporadic AD. This accelerated amyloidogenesis results from the triplication of chromosome 21, which contains the APP gene, leading to overproduction of the Aβ protein [26].

Recent neuroimaging studies have revealed that amyloid and tau pathology emerges in the middle to late 30s in DS, with a compressed timeline in which the amyloid and tau phases occur more rapidly than in sporadic AD, requiring targeting of both pathological proteins [24]. Positron emission tomography imaging effectively identifies the earliest stages of amyloid accumulation in DS, providing crucial biomarker evidence for treatment eligibility [24].

Turning to our neuropathological findings, CAA was preferentially and tightly associated with high-level ADNC. CAA was confined to DS patients who had reached Thal Aβ phase 5 and Braak NFT stage V–VI, and was absent in patient D6 despite abundant parenchymal Aβ deposition. This suggests that, even under lifelong APP overproduction, CAA in DS emerges only after the Aβ-tau cascade has reached advanced stages, consistent with progressive failure of perivascular Aβ clearance once parenchymal burden becomes maximal [15,24]. The uniform severity of CAA across the three DS+CAA patients (grade 2, stage 3, type 1, with spinal and bridging-vessel involvement) further indicates that DS, when it develops CAA, recapitulates the severe end of the sporadic CAA spectrum [15,26]. Furthermore, all three DS patients aged ≥ 48.7 years exhibited CAA, compared with only two of five elderly controls over 70 years. These findings suggest that CAA develops earlier and more consistently in DS than in cognitively intact aging, in keeping with accelerated aging-related neuropathology in DS. These inter-pathology associations are exploratory given n = 9, but the all-or-none character of CAA above and below the high-level ADNC threshold is sufficiently clear to warrant consideration in future biomarker and therapeutic studies in DS.

Beyond CAA, other aging-related pathologies in DS showed distinct patterns relative to ADNC. GVD co-progressed with NFT pathology, mirroring the established GVD-tau association in sporadic AD [41]. In contrast, LBD behaved as an independent co-pathology [27]. The absence of AGD, PART, ARTAG, and LATE-NC across all DS patients suggests that the DS neurodegenerative trajectory is largely confined to the APP-driven Aβ-tau axis and its downstream cerebrovascular and granulovacuolar consequences, without divergence into the other age-related proteinopathies common in cognitively normal elderly [8,23,31,34]. Pallidal mineralization in patients D7–D9 (aged 48.7 years and older) is consistent with the long-recognized association of this vascular alteration with DS [40,48]. In our cohort, similar mineralization was also detected in one elderly control (C14, 89.5 years) and one sporadic AD case (AD2, 83 years), both substantially older than our DS+CAA cases. This pattern indicates that pallidal mineralization is not specific to DS, but emerges approximately three to four decades earlier in DS than in non-DS individuals, in keeping with previous descriptions [40,48]. We adopt the term *mineralization* rather than *calcification* in light of recent evidence [39] that these deposits contain both calcium and iron. In our cohort, comparable mineralization was not detected in the cerebellar white matter or dentate nucleus, in contrast to the cerebellar arteriolar mineralization recently described by Szalardy et al. [39]; this discrepancy may reflect our small sample size (n = 3 DS+CAA), differences in CAA severity, or sampling variation. Together, these observations indicate that aging-related neuropathological changes in DS follow heterogeneous patterns: some (CAA, GVD, pallidal mineralization) appear to develop in parallel with or earlier than in age-matched controls, whereas others (AGD, PART, ARTAG, LATE-NC) are largely absent, suggesting that the DS aging trajectory is selectively channeled into specific pathological streams.

A particularly striking feature of our cohort was the universal attainment of Thal CAA stage 3 in all DS+CAA patients, in contrast to a maximum of stage 2 in CAA-positive sporadic AD and elderly control cases. Stage 3 — defined by involvement of the basal ganglia, thalamus, and lower brainstem — represents the most advanced and least frequent topographical extension of CAA in the original description by Thal et al. [42] and is observed only in a minority of high-grade ADNC cases. This convergence at stage 3 despite a younger age range than the sporadic AD group indicates that DS-CAA is not merely earlier in onset but also topographically more extensive, likely reflecting lifelong APP overexpression and saturation of perivascular Aβ clearance into deep penetrating arteries [15,26]. These findings are fully concordant with the recent autopsy study by Szalardy et al. [39], in which 10 of 11 adult DS cases also reached Thal stage 3, with frequent capillary (type 1) involvement and severe cerebellar leptomeningeal involvement (Vonsattel grade 2) — features uniformly reproduced in our DS+CAA cases. This convergence supports universal stage 3 progression as a reproducible hallmark of DS-CAA. With respect to compartment-specific Vonsattel grading, however, our results only partially aligned with Szalardy et al. [39]. While eight of their 10 cases reached neocortical leptomeningeal grade 3, the maximum grade in our cohort did not exceed grade 2 in any compartment, and in the neocortex, parenchymal vessels equaled or exceeded leptomeningeal vessels in two of three cases (D7 and D9). This divergence likely reflects the small size of our DS+CAA group (n = 3) and possible differences in age distribution and cortical regions sampled, both of which limit resolution for compartment-specific comparison. The Thal type 1 predominance shared across cohorts likely reflects the Aβ42 dominance characteristic of lifelong APP overexpression in DS [15,26]. Of note, Szalardy et al. [39] also reported occasional brain regions in which capillary CAA was present in the absence of concurrent arteriolar or venular involvement. In our cohort, no such capillary-only pattern was identified; capillary CAA consistently co-occurred with arteriolar and/or venular involvement, possibly reflecting the smaller size of our cohort or differences in regional sampling. The extension to spinal arteries and subdural/subarachnoid bridging vessels documented in our series should be interpreted with caution, as these sites are seldom included in standard autopsy protocols. Their report of hemorrhagic alterations without gross hematomas in 5 of 10 DS-CAA cases, together with our observation of SDH in two of three DS+CAA cases, further supports the link between advanced DS-CAA and hemorrhagic complications. Group-wise statistical comparisons (**Table 3**) confirmed complete between-group separation for Thal CAA stage 3 attainment, although the small group sizes precluded conventional significance (p = 0.100, the lowest possible exact two-sided p-value for this dataset). Together, these convergent observations support the view that severe, topographically extensive CAA with capillary involvement is a characteristic feature of advanced ADNC in DS.

Building on this comparison, the CAA-restricted occurrence of SDH in our cohort (2/3 DS+CAA vs. 0/6 DS without CAA), together with the histological observation of Aβ deposition in subdural bridging vessels, is concordant with population-level studies linking sporadic CAA to SDH risk [33] and with the recent autopsy report by Szalardy et al. [39]. Notably, the four SDH events documented in our control cohort (C8, C10, C12, C14) were all clinically traumatic in origin and occurred in CAA-negative individuals, providing a within-cohort contrast: in the absence of CAA, SDH in our series was attributable to identifiable trauma, whereas in DS+CAA patients SDH occurred without documented trauma despite the presence of severe vascular Aβ deposition. This pattern is consistent with — though does not prove — a CAA-related mechanism for the SDH events in DS. It should also be noted that our control cohort consisted of forensic autopsy cases, in which traumatic events such as falls, traffic accidents, and other injuries are over-represented compared with the general population. The relatively high frequency of traumatic SDH among controls (4/14) likely reflects this selection characteristic of forensic series, rather than the background prevalence of SDH in the general elderly population. This forensic-cohort context further strengthens the contrast between the SDH observed in CAA-negative controls — for which a traumatic etiology was clinically established — and the trauma-undocumented SDH events in our DS+CAA patients. However, as detailed in the Results, the marked cerebral atrophy in both SDH cases, the inability to fully exclude antecedent head trauma, and the small number of DS+CAA cases (n = 3) preclude any causal inference; a definitive CAA-SDH link in DS would require larger prospective autopsy series. Since sporadic CAA itself confers increased SDH risk [33], our findings should be interpreted not as a DS-unique association but as a hypothesis that lifelong APP overexpression may amplify SDH risk in DS — a consideration relevant to the vascular safety of emerging anti-amyloid therapies [26].

The absence of lobar ICH, cortical superficial siderosis, and lobar cerebral microbleeds in all nine DS patients of our cohort warrants discussion in the context of the long-standing debate on the hemorrhagic risk of DS-CAA. As reviewed by Buss et al. [6], lobar ICH is strikingly rare in DS despite severe vascular Aβ deposition — in marked contrast to APP-duplication kindreds, in whom ICH affects approximately one-third of carriers — leading to the hypothesis that DS may be relatively protected from CAA-related macrohemorrhage. Recent in vivo SWI-MRI studies have shown that lobar microbleeds and cortical superficial siderosis do occur in adults with DS with a posterior, lobar predominance consistent with CAA, but gross lobar ICH remains uncommonly reported [1,7]. Our autopsy findings align with this pattern: despite the severe CAA phenotype described above, no lobar ICH or microhemorrhagic markers were identified, and the only documented hemorrhagic events were the SDH episodes in D7 and D9. This is concordant with Szalardy et al. [39], who detected hemorrhagic alterations in five of 10 DS-CAA cases but no gross hematomas. Together, these observations suggest that the hemorrhagic phenotype of DS-CAA may be dominated by microhemorrhagic and extracerebral (subdural/bridging-vessel) changes rather than classical lobar parenchymal ICH. We therefore present our SDH findings not as evidence of a generally elevated ICH risk in DS, but as a compartment-specific observation to be considered alongside the predominantly negative ICH literature when evaluating the vascular safety of anti-amyloid therapy in this population.

In terms of broader therapeutic implications, two anti-amyloid monoclonal antibodies — lecanemab [46] and donanemab [36] — have received traditional FDA approval for early Alzheimer's disease, and both share safety considerations particularly relevant to DS. A recent investigation of lecanemab in DS brains demonstrated effective binding to SPs in all DS brains examined, but also extensive binding to CAA-affected leptomeningeal and cortical vessels [26], and donanemab has likewise been shown to bind cerebral amyloid angiopathy fibrils [37], indicating that vascular amyloid binding is a class-wide property. The clinical consequence is amyloid-related imaging abnormalities (ARIA), encompassing both ARIA-E (edema/effusion from blood-brain barrier compromise) and ARIA-H (microbleed/superficial siderosis from microvascular wall fragility), both mechanistically driven by antibody engagement of CAA-affected vessels [38]. Our findings, together with those of Szalardy et al. [39], indicate that severe, topographically extensive CAA — including capillary, spinal, and bridging-vessel involvement — is essentially universal in adults with DS who have reached advanced ADNC. This near-universal severe CAA represents a substantial limitation for anti-amyloid antibody therapy in DS, since the pathological substrate defining treatment indication is also the substrate of greatest ARIA risk. DS-specific clinical trials with prospective ARIA-E and ARIA-H monitoring and pre-treatment CAA characterization will be essential to establish the benefit-to-risk balance of anti-amyloid therapy in this population.

Individuals with DS also face abnormal accumulation of phosphorylated tau in the early stage of life [12]. Plasma p-tau217 accurately predicts brain amyloid pathology in DS patients [20,35]. Current therapeutic approaches include the use of tau-targeting antibodies (JNJ-63733657 and BMS-989446) and vaccines (ACI-35) in clinical trials [13,17,21]. However, DS individuals were historically excluded from major AD trials, generating data gaps [3]. Future research must expand beyond amyloid-targeting to include tau pathology, neuroinflammation, and synaptic dysfunction mechanisms through DS-specific clinical trials with adapted assessments [32].

Despite providing valuable insights into neuropathological aging patterns in DS patients, our study has several limitations. The small sample size of nine patients, while representing a substantial collection given the rarity of DS autopsies, limits the statistical power for comprehensive analyses. The exclusive Japanese population, while ensuring genetic homogeneity, may limit generalizability across ethnic groups. Additionally, the cross-sectional approach, although it reveals important age-related patterns, cannot capture individual longitudinal progression dynamics. Furthermore, our cohort spans a 49-year autopsy collection period (1973–2022), during which medical care and living environments for individuals with DS in Japan changed substantially. This temporal heterogeneity may introduce a cohort effect that should be considered when interpreting the age at which ADNC emerges in our series. Limited clinical documentation restricts detailed clinicopathological correlations. The image analysis comparison between DS+AD (n = 3) and sporadic AD (n = 3) and the group-wise statistical comparisons of CAA scores are all exploratory; small group sizes preclude formal statistical inference and impose inherent floors on exact p-values, even when complete categorical separation is observed. Quantitative differences should therefore be interpreted as supportive rather than confirmatory of the morphological characterization. The clustering of SDH within DS+CAA cases is observational; the retrospective design, the limited availability of head trauma history, and the marked cerebral atrophy in both SDH cases preclude attribution of SDH to CAA.

This neuropathological study of nine autopsied DS patients revealed that all four patients aged 28 years and older exhibited moderate to severe ADNC, whereas no age-range-matched control showed comparable findings. Given the small sample size, these findings should be interpreted descriptively. Characteristic unclassifiable SPs and severe CAA — including in the spinal arteries and subdural bridging vessels — were notable features in this DS series, with implications for the vascular safety of anti-amyloid therapies, while the absence of other aging-related proteinopathies (AGD, PART, ARTAG, LATE-NC) suggests selective channeling of the DS aging trajectory into the APP-driven Aβ-tau axis. These findings provide crucial insights into AD pathogenesis and emphasize the importance of developing targeted therapeutic strategies while considering safety implications.

## Ethics approval statement

This study was performed in compliance with the principles of the Declaration of Helsinki. Approval was obtained from the institutional review boards of Aichi Medical University. Signed informed consent for autopsy, the archiving of tissue for research purposes, and genetic analysis were obtained from the family members of all the patients in compliance with the Ethical Committee for Medical Research of Aichi Medical University.

## Data availability statement

The data that support the findings of this study are available from the corresponding author upon reasonable request.

## Author contributions

HMi conceptualized and prepared the original draft of the manuscript; KY and HMi performed the histopathological experiments; DT, NT, HMo and HMi assembled the clinical information; YH, YR, AA, JS, HH, AK, YI, and HMi performed the histopathological analysis; HMi visualized the data; and all authors reviewed, edited, and approved the manuscript.

## Declaration of generative AI

No generative AI or large language model (LLM) tools were used in this study.

## Conflict of interest statement

The authors declare that they have no conflicts of interest.
